# Experiences of Aging with Opioid Use Disorder and Comorbidity in Opioid Treatment Programs: A Qualitative Analysis

**DOI:** 10.1007/s11606-024-08676-z

**Published:** 2024-03-04

**Authors:** Benjamin H. Han, Mirella A. Orozco, Mari Miyoshi, Heidi Doland, Alison A. Moore, Katie Fitzgerald Jones

**Affiliations:** 1 Division of Geriatrics, Gerontology, and Palliative Care, Department of Medicine, University of California San Diego, La Jolla, CA USA; 2 Department of Psychiatry, University of California San Diego, La Jolla, CA USA; 3 New England Geriatric Research Education and Clinical Center (GRECC), VA Boston Healthcare System, Jamaica Plain, MA USA

**Keywords:** aging, opioid use disorder, methadone

## Abstract

**Background:**

The number of older adults entering opioid treatment programs (OTPs) to treat opioid use disorder (OUD) is increasing. However, the lived experiences of aging in OTPs have not been examined.

**Objective:**

To explore the aging experience with OUD and barriers to medical care for older adults who receive care in OTPs.

**Design:**

From November 2021 to July 2022, we conducted 1-to-1, semi-structured qualitative interviews in English and Spanish, audio-recorded, transcribed, systematically coded, and analyzed to identify key themes regarding the challenges of aging with OUD and managing chronic diseases.

**Participants:**

Thirty-six adults aged ≥ 55 enrolled in OTPs in San Diego, California.

**Approach:**

A descriptive qualitative approach was used. Major themes and subthemes were identified through thematic analysis until thematic saturation was reached.

**Key Results:**

All participants were on methadone and had a mean age of 63.4 (SD 5.1) years; 11 (30.6%) identified as female, 14 (39%) as Hispanic/Latino, and 11 (36%) as Black, with a mean duration of methadone treatment of 5.6 years. Chronic diseases were common, with 21 (58.3%) reporting hypertension, 9 (25%) reporting untreated hepatitis C, and 32 (88.9%) having ≥ 2 chronic diseases. Three major themes emerged: (1) avoidance of medical care due to multiple intersectional stigmas, including those related to drug use, substance use disorder (SUD) treatment, ageism, and housing insecurity; (2) increasing isolation with aging and loss of family and peer groups; (3) the urgent need for integrating medical and aging-focused care with OUD treatment in the setting of increasing health and functional challenges.

**Conclusions:**

Older adults with OUD reported increasing social isolation and declining health while experiencing multilevel stigma and discrimination. The US healthcare system must transform to deliver age-friendly care that integrates evidence-based geriatric models of care incorporated with substance use disorder treatment and addresses the intersectional stigma this population has experienced in healthcare settings.

**Supplementary Information:**

The online version contains supplementary material available at 10.1007/s11606-024-08676-z.

## INTRODUCTION

In recent decades, drug overdose deaths among older adults have quadrupled, with significant health disparities experienced among minoritized older populations.^1,2^ Substance use disorders (SUDs) among older adults are expected to continue to rise because of shifting demographics, with the Baby Boomer generation having higher rates of substance use than preceding generations and age-related health challenges that may drive substance use, including trauma emergence, late-life stressors, or chronic pain.^3^ There have also been sharp increases nationally in the number of older adults entering opioid treatment programs (OTPs) for opioid use disorder (OUD) treatment.^4,5^ This growing population of older adults with OUD has a substantial burden of chronic disease and often receives fragmented care with high rates of hospitalization.^6,7^ Despite the increasing need for services tailored to the needs of older adults with OUD, less than a quarter of programs offer specialized care aimed at older populations.^4^

The benefits of methadone to reduce mortality and improve quality of life among people with OUD are well documented.^8–10^ However, as people age, methadone may be associated with new health-related challenges, including falls^6^ and obstacles to access and safe use.^11^ For example, current federal policy dictates methadone for the treatment of OUD can only be delivered through OTPs that often require long commutes and frequent visits, which can be challenging for older adults.^12^ OTPs are also currently segregated from the rest of the healthcare system, rarely providing primary or specialty level care and limiting treatment to OUD.^12^ Indeed, methadone is currently not visible on state prescription drug monitoring programs and is often absent from the medical record.^13^ Consequently, clinicians outside of OTPs may be unaware of their patients’ OUD history or methadone treatment. Likewise, the current OTP system may be ill-equipped to support older adults with OUD who are medically and socially complex.^14^

There is a recent national initiative to improve the care of older adults by creating age-friendly care health systems that focus on evidence-based principles of high-quality geriatric care (known as the “5Ms” matters most, medication, mentation, mobility, and multicomplexity).^15,16^ This presents an important opportunity to improve the health of older adults with OUD by addressing care in these five domains. For example, what “matters most” can include aligning care with older adults’ goals and preferences both regarding health and substance use goals, “medications” acknowledges age-related changes that can increase the risk for side effects from methadone or other medications, “mentation” includes screening older adults with OUD for mood and cognition changes, “mobility” refers to optimizing function such as screening for falls, and “multicomplexity” includes integrated care for multiple chronic conditions including OUD.

As a first step to developing age-friendly OUD models of care, we sought to explore the current experiences of aging with OUD and barriers to chronic disease management for older adults who receive care in OTPs. In designing this study, we considered that inequities exist across a range of chronic diseases and disease-related complications by race and ethnicity due to long-standing barriers in health care rooted in structural racism, which has resulted in a segregated treatment landscape between methadone in OTPs and office-based buprenorphine.^17–19^ For older adults with OUD, systemic injustices have often compounded over decades.^20^ Further, methadone policies are changing with increased use of take-home dosing. Therefore, study findings may have important implications on how to best inform models of geriatric-based care consistent with the 5Ms to help achieve health equity and treatment flexibility for this population made vulnerable through a lifetime of health disparities.

## METHODS

### Setting and Study Population

The inclusion criteria for this study were age 55 and older and enrolled in one of two large OTPs in central San Diego, California. The qualitative interviews for this study were conducted between November 2021 and July 2022. A descriptive phenomenological qualitative design was selected to illuminate the lived experience of aging with OUD with deliberate attempts to minimize biases and preconceptions through bracketing.^21^ Specifically, descriptive phenomenology aims to understand how individuals with shared characteristics experience a phenomenon, that is, how older adults experience aging with OUD. We used purposeful sampling recruiting of individuals who were older adults currently enrolled in OTPs that could describe the experience of aging with OUD in either English or Spanish. We defined older adults as age ≥ 55 years old because adults with OUD can experience premature aging and experience early onset of geriatric conditions.^6^ The OTP staff, including social workers, peer counselors, and nurses, assisted with participant identification. After completion of informed consent, all interviews were conducted in a private room at the OTPs. Recruitment ended when no new themes emerged consistent with thematic saturation.^22^ The manuscript follows the Standards for Reporting Qualitative Research (SRQR) guidelines.^23^ The study was approved by the University of California San Diego institutional review board and Behavioral Health Services for the County of San Diego.

### Data Collection

The principal investigator (B.H.H.), board-certified in geriatric and addiction medicine and trained in qualitative methods, conducted in-person, semi-structured qualitative interviews in English in a private room. Interviews in Spanish were conducted by a trained research associate (M.A.O.) and supervised in the room by the principal investigator (B.H.H.). None of the participants were under the clinical care of the principal investigator. A survey (Supplemental Appendix 1) was used to collect self-identified demographic information, including age, racial and ethnic identity, gender identity, insurance status, medication for OUD type, duration of care at the OTP, and self-reported presence of chronic diseases. The interview guide (Supplemental Appendix 1) was created in English and Spanish by the study team and broadly focused on (1) the experience of aging with OUD, (2) interactions with the healthcare system (related to geriatric conditions and age-friendly care), (3) management of OUD and other health-related challenges, and (4) perceived barriers to receiving care for age-related conditions. Interviews lasted approximately 45 min, were audio-recorded, and transcribed verbatim. Participants were provided $30 compensation for their participation.

### Data Analysis

Interview transcripts were imported into Atlas.ti, version 8 (Scientific Software Development GmbH). Coding and analysis were performed according to the principles of descriptive qualitative analysis research and a priori thematic analysis.^24,25^ Three investigators (M.A.O., M.M., H.D.) independently reviewed and coded the transcripts. Preliminary codes were reviewed and consolidated into a final codebook (Supplemental Appendix 2). We used a seven-step modified Colaizzi method^25^ for data analysis to enhance trustworthiness: (1) read the transcripts several times to get a broad understanding, (2) met as a group to identify key codes related to the research question, (3) combined the coded quotations and confirmed accuracy, (4) analyzed and grouped quotations to identify major themes and create a code book, (5) reviewed major themes using the constant comparative method and engaged in active discussion to resolve discrepancies, (6) review transcripts to validate themes iteratively alongside interview data, and (7) conducted multiple discussions until consensus was achieved.^22,26,27^

## RESULTS

Thirty-six individuals participated in this study, all receiving methadone for OUD and none receiving buprenorphine or naltrexone. Their mean age was 63.4 (SD 5.1) years with an age range of 55 to 77 years; 11 (30.6%) identified as female, 14 (39%) identified as Hispanic or Latino, and 11 (36%) identified as Black; and the mean duration of current methadone treatment was 66.9 months with a range of 1 to 240 months. Chronic diseases were common, with 21 (58.3%) reporting hypertension, 9 (25%) reporting untreated hepatitis C, and 32 (88.9%) having two or more chronic diseases (Table 1). Three major themes and several subthemes emerged (Table 2) on the experience of aging with OUD and barriers to medical care for older adults who receive care in OTPs: (1) avoidance of medical care due to multiple intersectional stigmas, including those related to drug use, SUD treatment, ageism, justice involvement, and housing insecurity; (2) increasing isolation with aging and loss of family and peer groups; (3) the urgent need for integrating medical and aging-focused care with OUD treatment in the setting of increasing health and functional challenges.

**Table 1:** Demographic and Chronic Disease Characteristics of 36 Participants

Characteristic	No. (%)
Age, mean (SD), years	63.4 (5.1)
Age range, years	55–77
Gender identity	
Male	25 (69.4)
Female	11 (30.6)
Race/ethnicity	
White	7 (20.0)
Black/African American	11 (30.6)
Hispanic/Latino	14 (39.0)
More than one race or Native American/Hawaiian	4 (11.0)
Prefer interview done in Spanish	4 (11.0)
Health insurance	
Medicare	14 (39.0)
Medicaid	36 (100.0)
Medication for opioid use disorder	
Methadone	36 (100%)
Current duration of receiving care from opioid treatment program	
Mean (SD), months	66.9 (64.2)
Range, months	1–240
Chronic diseases	
Hypertension	21 (58.3)
Osteoarthritis	14 (38.9)
Diabetes	13 (36.1)
Active hepatitis C	9 (25.0)
Neuropathy (spinal stenosis, diabetes-related)	9 (25.0)
Anxiety disorder	8 (22.2)
Coronary artery disease	7 (19.4)
Chronic obstructive pulmonary disease	5 (13.9)
Major depression	5 (13.9)
Schizophrenia	4 (11.1)
Cancer (includes breast, colon, liver)	3 (8.3)
Cirrhosis	3 (8.3)
Congestive heart failure	3 (8.3)
Peripheral arterial disease	2 (5.6)
Obstructive sleep apnea	1 (2.8)
Osteoporosis	1 (2.8)
2 or more of the above chronic diseases	32 (88.9)

**Table 2:** Major Themes and Subthemes Summarizing the Experience of Aging with Opioid Use Disorder (OUD) Within Opioid Treatment Programs (OTPs)

*Theme 1: Avoidance of medical care due to intersectional stigma* *Subthemes* • Experienced discrimination because of their drug use • Experienced discrimination because of being on methadone or receiving care with an OTP • Experienced discrimination due to lack of housing • Experienced discrimination due to history of incarceration • Anxiety about disclosing OUD with healthcare providers, preferring to not disclose • Reliance on emergency department care • Delaying care because of stigma
*Theme 2: Increasing isolation with aging and loss of family and peer groups* *Subthemes* • Housing insecurity challenges • Loss of peer groups and family who have died and others with estrangement from family • Lack of peer social supports with age including concerns about needing a caregiver or assistance with instrumental activities of daily living (IADLs) • Loss of friends from drug overdoses • Increase loneliness after cessation of drug use and as social networks change • OTP structure (i.e., need for frequent visits) prevents other daily or social activities
*Theme 3: The urgent need for integrating medical and aging-focused care with OUD treatment in the setting of increasing health and functional challenges* *Subthemes* • Aging-related challenges: nutrition, cognitive changes, anxiety for the future, incontinence, vision, chronic pain, declining physical health, mobility, and need for assisted devices • Transportation challenges • Reliance on emergency department care • Anxiety that methadone can affect aging and overall health and chronic disease long-term • Perception that integrated medical care with OUD treatment would be a benefit with aging and managing other chronic diseases • Lack of provider communication • Better need for mental health providers at OTPs • More information about dealing with getting older needed • Perceived need to address aging in one setting • Perceived need to stop methadone as one ages • Variable relationships with primary care providers

### Theme 1: Avoidance of Medical Care Due to Intersectional Stigma

Participants, as they aged, frequently sought healthcare outside the OTP but encountered discrimination, causing them to make “a game-time decision” whether to disclose substance use. They described being treated like a “dog” and “dirt” and needing to remind clinicians, “I am a person like you.” Despite a sense that they required more medical care as they aged, participants described mistrust of the medical system, causing them to delay routine healthcare or rely on the emergency department. Participants also noted several intersectional and converging factors they perceive drive poor treatment in the medical system. These include ageism, drug-related stigma, and pejorative beliefs surrounding housing insecurity or justice system involvement. Participants described a sense that the stigma surrounding aging with substance use was pervasive.

“*They see you come in homeless, they look down on you. They see you come in on drugs, they look down on you. They see you are old and do drugs and they look down on you more.*”

Participants felt clinicians perceived their drug use as a willful poor choice. Implying substance use was something participants should have outgrown as they aged and was to blame for many of their health-related problems.

“*When I go to the hospital once they know I am a user, they treat me very different…They talk about that you use heroin and they treat you worse than a dog and when you say you are in pain, they say, ‘it is because of the drugs’ and ‘you should not use that.’*”

For many participants, they avoided or did not disclose their SUD history because of their experience of discrimination. They reported receiving sub-standard care following past disclosures. Participants described, “when I don’t tell them it is a lot better.”

“*Everything was fine…until they found out I was using drugs. And I got treated different. You know? So, I’m kind of reluctant to even share any of that with these doctors or whoever they are ‘cause yeah, I start getting treated way different… Stereotyped me ... I’m a human being like anybody else.*”

### Theme 2: Increasing Isolation with Aging and Loss of Family and Peer Groups

Cumulative losses increased isolation as participants aged. Many participants voiced their inner circle of support becoming smaller and life increasingly revolving around their methadone treatment and OTP attendance.

“*I realize, most of my friends, family, associations… have passed away, my brother and stuff. As I get older, I realize it is just me and methadone that’s left.*”

They described thinking about their own mortality, which sometimes motivated their commitment to improving their well-being.

“*I’m at a kind of a crossroads right now at 58 years old. Am I gonna ignore it? Am I going to seek to live for another 10 or 15 years or whenever? Or am I gonna continue on my path and probably be dead... If I make it to 65, it’d be a miracle. Everyone I was close to is already gone.*”

Participants wanted more mental health support within the OTP because it was hard to relate to others and share their stories or experiences of traumatic events.

“*It’s at this point in life you want to hold a conversation with no one…I didn’t want to hold a conversation with anybody. I’ve been through a lot. my parents had passed, [they] were on drugs. My brother passed first. Then my dad, then my mom. And I normally don’t talk about that because it makes me upset.*”

### Theme 3: The Urgent Need for Integrating Medical and Aging-Focused Care with OUD Treatment in the Setting of Increasing Health and Functional Challenges

Participants described the unique challenges of getting older with OUD, including impairments in mobility and vision, falls, cognitive changes, housing and food insecurity, and declining physical health. Participants had concerns that chronic symptoms were not addressed, including chronic pain and shortness of breath due to chronic diseases that may worsen with age.

“*Something that worries me about being 55 years old is that now that I am older, my system is failing me more… I need more help for my health than before.*”

Participants noted transportation as a significant barrier to engagement in other healthcare, with OTP attendance being prioritized. Most did not have access to a vehicle and relied on public transportation. The lack of transportation or unreliable transportation limited their ability to seek regular care and engage in specialty care.

“*For many of us, our day consists of chasing down the bag. Now, even if we are not using, it is still a very big challenge to get here. Transportation wise…and we don’t have time to go see a doctor or go to a free clinic. If there was [services at the OTP]… we could kill two birds with one stone. That would be great, especially for the diabetes and getting older….*”

Participants described wanting care while they were already interfacing with a clinician.

Waiting to come in next week, or tomorrow, it really does not work.

One participant said it would be “one hundred percent better” to receive care in one location—especially given their increasing need for assistive devices such as walkers, wheelchairs, and medical equipment, including oxygen tanks.

“*It would be a tremendous help to have more care here, especially getting older… We come here every day. And a lot of people have trouble getting to the doctor. Or they don’t go because it’s out of the way.*”

Many participants felt OTPs would be an ideal location for integrated care of chronic conditions, including primary care and subspeciality care, because it was familiar, and they had a social network and felt less judgment from people who know them and care for people with SUDs.

## DISCUSSION

Older adults living with SUD are overlooked in clinical care, research, and policy, and this descriptive qualitative study provides a deeper understanding of their lived experience of aging with OUD. This study identified important themes that have implications for integrated and age-friendly healthcare. First, any geriatric care model for older adults with SUD must address stigma and ageism. Some of the experiences of our cohort concerning stigma are well documented in the OUD literature;^28^ however, this study highlights that intersectional stigma, including ageism, is a fundamental hindrance to age-friendly care among people with OUD receiving methadone. Intersectional stigma is defined as a synergistic effect of various forms of oppression that, when combined, create distinct social and structural inequities.^29^ Interventions that reduce intersectional stigma begin with dismantling oppressive power structures that fuel stigma, including SUD treatment systems that are not set up to address age-related changes, such as changes to mobility and cognition that may make regular attendance challenging. Alternative models for delivering methadone, including mobile methadone units, telehealth, or community-based pharmacies, can reduce barriers for older patients with mobility impairment or who are homebound or develop cognitive impairment requiring assistance with take-home doses. This may also decrease stigma related to SUD and aging by normalizing OUD as a chronic condition deserving of health care and social inclusion, as it is not delivered in a separate, often stigmatized location.^30–32^

Additionally, participants in our study described disincentives to disclose their substance use history, social determinants of health (criminal justice involvement or housing insecurity), and methadone treatment, fearing it would result in worse treatment and medical care. A key approach of geriatric medicine is balancing multicomplexity, and if substance use is not disclosed, age-friendly care will be challenging to achieve. It is important to recognize that for older adults, these experiences often have compounded over decades and are experienced directly by the healthcare system, which creates barriers that prevent people from accessing care.^33^ The study findings demonstrate that stigma reduction efforts must be “intersectional” and address ageism.^29,34,35^ The participants, the majority who identified as Black or Hispanic/Latino, described being victims of several ageist beliefs, such as being told that they should outgrow their substance use coupled with a misconception that OUD is a willful choice. This may further reflect the structural racism that results in who has access to office-based buprenorphine while disproportionally Black, Indigenous, and People of Color receive treatment through more restrictive and often punitive OTP settings.^17,18^

Social isolation and loneliness are commonly experienced by older populations,^36^ and this was echoed by research participants. This population has experienced significant loss of peers and family members, many through overdoses, which depleted social networks. The intersectional stigma associated with SUD and age could limit new interactions or prevent the development of social networks. The lack of social connectedness could have profound negative effects on the health of this population, including an increased risk for mortality, cognitive decline, a return to drug use, use of psychoactive substances, and decreased treatment engagement.^36–38^ Consideration should be given for examining existing interventions, including group sessions, psychosocial supports, and cognitive behavioral therapy, to reduce social isolation and loneliness among older adults in OTPs for this population.^39^

Despite commonly endorsing social isolation and loneliness, some participants described finding social connection in OTPs; thus, receiving methadone through this space may have an important role for some patients. Still, moving methadone treatment more within healthcare systems or bringing geriatric care to OTPs could enhance care by improving medication management and minimizing potential inappropriate medications or drug-drug interactions.^40^ The participants in the study also highlighted the need for minimally disruptive care^41^ for OUD and other chronic diseases, for example, coordination of care between OUD care and primary care or expanding healthcare settings where methadone can be delivered to overcome transportation barriers, mobility issues, and fear of stigmatization. One result of this is a quarter of participants had active hepatitis C and never received curative treatment despite a range of models of care to deliver hepatitis C management for this population.^42,43^

While we did not collect specific details about housing, a common theme in the interviews focused on the challenges of housing insecurity, especially related to added stigma and lack of integrated care. In California, people experiencing housing instability are aging with an overrepresentation of minoritized groups,^44^ which is a population that experiences accelerated aging with a high rate of geriatric conditions, cognitive impairment, and SUDs.^45,46^ Therefore, integrated care models for this population must also consider housing instability and ways to provide additional support, including multidisciplinary intensive case management focusing on housing and outreach.

While many studies have examined integrated care models for patients with SUDs, they focus on younger patients or do not emphasize geriatric-focused health outcomes.^47–50^ Meanwhile, while there is a wealth of literature on the effects of geriatric interventions for vulnerable older populations that show improvements in mortality, nursing home use, and quality of care in various settings, none has been studied among older adults with SUD.^51–53^ The results from this study emphasize the pressing need to provide older adults with OUD with integrated geriatric-based models of care within substance use treatment settings. For example, coordination between OTPs and specialists such as geriatric and palliative care clinicians can help support attention to common comorbidities such as chronic pain, thereby avoiding methadone interruptions.^54^ Further, focusing on harm reduction and shared decision-making that recognizes that substance use is biologically and socially complex with inherent inequities is critical in settings where older adults receive care, such as hospitals.^55^ Including members of the geriatric workforce in SUD treatment settings could lead to greater attention to geriatric conditions such as falls or cognitive impairment voiced by many participants. In turn, this can lead to dialogue around alternatives to OTP-based methadone treatment, such as office-based buprenorphine or thoughtful consideration of deprescribing or de-escalating methadone if burdens outweigh benefits. Fortunately, the principles of geriatric care and harm reduction align with patient-centered care.^56^ Future studies should also examine changes in perspectives of aging with OUD by age decade (e.g., 50 s compared to 70 s) as well as patients with co-occurring SUDs.

### Limitations

Our study has important limitations. First, the findings presented are from patients who receive care from just two OTPs in one city and given that the services provided in OTPs can vary widely throughout the country, the experiences are not generalizable to older patients who receive care from other OTPs. Second, our study sample was under-represented by people identifying as female. Third, all participants were receiving methadone for OUD, and none were receiving buprenorphine or naltrexone, which largely reflects the treatment population in the two OTPs we recruited from, as well as clinical practices in OTPs throughout the country.^57^ Additionally, our interviews did not specifically probe about knowledge, experience, or access to buprenorphine, and therefore, our analysis is unable to fully explore how participants could view the use of buprenorphine on the challenges faced by this population. Fourth, most participants had long-term OTP treatment histories, which reflects the treatment population from the two OTPs and may not reflect the experiences of older patients entering OUD treatment for the first time. However, we were able to recruit a demographically diverse population, and consistent with the goal of qualitative research, we focused on deep understanding rather than generalizability.

## CONCLUSIONS

In this qualitative analysis, older adults with OUD reported increasing social isolation and declining health while experiencing multilevel stigma and discrimination living with multimorbidity and unmet healthcare needs. Amidst the public health crisis of older adults dying from drug overdoses, we must transform our current healthcare system and eliminate isolated models of SUD treatment to enable greater holistic, integrated care across the age continuum. Older adults with SUD living with comorbidity must have access to age-friendly, evidence-based geriatric and addiction medicine treatment wherever they receive care, and the integration of these specialties is what would work best for many patients.^58^ Furthermore, such age-friendly integrated care must recognize the lifelong and intersectional stigma this population has experienced, especially in healthcare settings.

## Supplementary Information

Below is the link to the electronic supplementary material.

Supplementary file1 (DOCX 25 KB)
