# Supplementary material for: Experiences of Aging with Opioid Use Disorder and Comorbidity in Opioid Treatment Programs: A Qualitative Analysis
Source: J Gen Intern Med. 2024 Mar 4;39(9):1673–80. doi: 10.1007/s11606-024-08676-z (PMC11255154; doi:10.1007/s11606-024-08676-z)
Supplement: Supplementary file 1 — Supplementary file1 (DOCX 25 KB) [file 11606_2024_8676_MOESM1_ESM.docx]

**Appendix 1: Interview Guide**

**Chronic Disease and Health Outcomes of Older Adults with Opioid Use Disorders**

**QUALITATIVE INTERVIEW GUIDE**

**V. 3.0 (11/2021)**

**Participant ID: _________ Interviewer Initials: _________ Clinical Site: _____.**

**Date: _____________**

**Opening statement:** Thank you very much for coming to the interview today. We would like to learn more about your experience with chronic medical diseases and how you manage them as a patient in this opioid treatment program. I am also interested in your experience as someone who is 55 years of age or older since your experience may be different from younger patients.

This interview is an opportunity for me to learn from you. I’m hoping that you will teach me about your own experiences and opinions as a patient – you are the experts here. You were asked to be interviewed for this study because all of you are a patient in this opioid treatment program. The focus here is not your substance use or history of use, but rather how you think that people age 55 and older with chronic diseases who receive care here can manage their health.

To get started, I am going to ask you some brief structured questions, and then we will move on to a more open discussion. These questions will ask about your age and other background characteristics. All answers you provide are confidential, as we discussed. Is it all right if we get started?

1. **How old are you?**
   1. ________ years old
2. **Do you consider yourself to be Hispanic or Latino?**
   1. Yes
   2. No
3. **How would you describe your racial identity?** *(read list; check all that apply)*
   1. American Indian or Alaska Native
   2. Asian
   3. Black or African American
   4. Native Hawaiian or Other Pacific Islander
   5. White
   6. More than One Race
   7. Other **(*Specify ___________________________*)**
4. **Would you describe yourself as:**
   1. Male
   2. Female
   3. Transgender Man
   4. Transgender Woman
   5. Genderqueer
   6. Other **(*Specify ___________________________*)**
5. **What health insurance do you currently have:**
   1. Medicare
   2. Medicaid (Medi-Cal)
   3. Private insurance
   4. VA Health Care
   5. Other
6. **Do you currently receive:**
   1. Methadone
   2. Buprenorphine
7. **How long have you been receiving care from this clinic (months)?**
8. **Please tell me of any chronic medical diseases have you been told you have?**

**INTRODUCTION**

**READ:** OK, now I’d like to move into the part of this study that is more like a conversation. As a reminder, there are no right or wrong answers to any of the questions I will ask today, so please feel free to speak openly and honestly.

I would also like to confirm that I can record the audio from our conversation today so I can focus on what you are saying without having to take too many notes. **Is it OK with you that I record the audio from this interview?**

**Do you have any questions before we begin?**

**[TURN ON RECORDER; READ]:** This is [interviewer] conducting a [participant number] interview with [P##] on [date] at [time] at [location].

What we are talking about today is the issue of aging and managing chronic diseases for people with opioid use disorder. Our goal is to better address managing your chronic diseases. We are interested in hearing what you think.

1. **To get started, can you tell me some challenges you are experiencing as you get older living with opioid use disorder?**

*Probes:*
*- Do you experience any discrimination either for your addiction treatment or getting older?*

- *What worries you as you get older living with a history of opioid use disorder?*

1. **As you age, are there specific concerns that you have related to your health?***Probes:*
   *- What health problems are most important to you right now?*
2. **(Spanish interviews only) What challenges do you have not being a native English speaker as it relates to your health and the care you receive here?**

*Probes:*
*- Do you find it difficult to access care that you fully understand?*

1. **Do you have a primary care provider or a source for regular medical care?**
   1. **If yes, how do you feel about your current relationship with your provider? Do you feel that your chronic medical diseases are currently well managed?**
   2. **If no, is there a particular reason why you do not have one? What barriers do you think there are for you to have a primary care provider?***Probes:*
      *- Do you feel comfortable with your primary care provider, especially when talking about your opioid use disorder?
      - Are you worried about what providers will think about you receiving care from an opioid treatment program?*

- *Have you avoiding receiving primary care in the past because of your history of opioid use disorder?*

*- Have you ever delayed seeking medical care because of your history of opioid use disorder?*

1. **As people get older, they tend to have more chronic medical problems and take more prescribed medications. In addition, many older adults are at higher risk for having what we call “geriatric conditions” such as falls, memory problems, urinary problems or have increasing difficulty with carrying out daily activities. If you have a provider, do you feel that they are addressing or would be able to address some of these issues if it were to come up?**

*Probes:*
 *- If not, why?*

*- If not, where would you go to have these issues addressed?*

1. **What matters most for you in managing your chronic medical problems?**

*Probes:*
 *- What do you want your healthcare to be able to do for you?*

1. **What are your thoughts about other services you currently receive from the opioid treatment program other than management of methadone (or buprenorphine)?**

*Probes:*
*- Do you feel this clinic is can or able to address any age-related needs that you may have?*

1. **Let’s say we were planning start offering services to manage chronic medical diseases, like diabetes or hypertension and other issues that may arise as you age specifically for patients 55 and older for patients in this clinic. What do you think is the single most important thing we need to consider before doing that?**

**WRAP-UP**

**READ:** Thank you for sharing your opinions and experiences with me.

**OK, is there anything else that you’d like to tell me about these topics?**

Thank you again for taking the time to talk with me. **[TURN OFF TAPE RECORDER]**

**Appendix 2: Codebook**

| Code (# of occurrences) | Description |
| --- | --- |
| Unique challenges of getting older with opioid use disorder (OUD): Mobility (23) | Many participants expressed concerns about increasing mobility issues. |
| Unique challenges of getting older with OUD: Falls (17) | Many participants expressed concerns about frequent falls including need to use assistive devices. |
| Unique challenges of getting older with OUD: Chronic pain (41) | Many participants talked about uncontrolled chronic pain and its impact on overall health, function including instrumental activities of daily living, and overall well-being. |
| Chronic pain not taken seriously (23) | Frustration that chronic pain is not being addressed either by opioid treatment programs (OTP) or outside providers. PCPs unwilling to address pain concerns. |
| Unique challenges of getting older with OUD: Social challenges (18) | Homelessness and housing insecurity was a major issue for some participants. Those with stable housing expressed concern for needing caregivers and assistance with activities of daily living. |
| Unique challenges of getting older with OUD: Declining physical health (10) | Concern over the increase in chronic diseases and symptoms including chronic pain, shortness of breath. |
| Unique challenges of getting older with OUD: Management of chronic diseases (21) | Concern over the lack of control for diabetes or high blood pressure and difficulty taking medications. |
| Unique challenges of getting older with OUD: Nutrition (16) | Concern over appetite and accessing proper nutrition and food. |
| Unique challenges of getting older with OUD: Cognitive changes (24) | Concern for memory and cognitive changes. |
| Unique challenges of getting older with OUD: Anxiety for the future (14) | Uncertainty about the future as someone who uses drugs and is getting older. |
| Unique challenges of getting older with OUD: Incontinence (11) | Concern for issues related to urinary incontinence not being addressed and shame about incontinence. |
| Unique challenges of getting older with OUD: Vision (10) | Concern for issues related to vision changes and vision loss. |
| Increasing social isolation (30) | Loss of peer groups and family who have died and others with estrangement from family. Lack of peer social supports with age. |
| Friends dying due to fentanyl (4) | Loss of friends from drug overdoses. |
| Increased loneliness after stop using drugs (6) | Social networks change once stopped using drugs and difficulty socializing when sober. |
| Transportation (22) | Most did not have access to a vehicle or drive and relied on public transit or others for transportation. Lack of transportation impacts receiving regular medical care and is largest barrier to accessing care. |
| Poor relationship with PCP (30) | Regarding lack of trust with medical provider or does not see them regularly. |
| Reliance on emergency department care (24) | Regarding avoiding or delaying care until needing to go to an emergency department. |
| Experienced stigma from healthcare settings: Drug use (34) | Experienced discrimination because of their drug use. |
| Experienced stigma from healthcare settings: Drug use treatment (30) | Experienced discrimination because of being on methadone or receiving care in opioid treatment programs. |
| Experienced stigma from healthcare settings: Homelessness (12) | Experienced discrimination due to lack of housing. |
| Experienced stigma from healthcare settings: Criminal legal involvement (15) | Experienced discrimination due to history of incarceration. |
| Experienced stigma from healthcare settings: Aging and drug use (6) | Experienced discrimination due to using drugs by an older adult. |
| Integrated services needed in opioid treatment program (34) | The need to address aging in the OTP setting. |
| Desire to quit methadone (14) | The need to stop methadone as one ages. |
| Language is not a barrier for receiving care (3) | Among Spanish-speakers, there did not appear to be any barriers in the OTPs or in other healthcare settings for accessing care. |
| Avoidance of discussing history of opioid use (17) | Participants anxious about disclosing OUD with healthcare providers. |
| Methadone side effects (37) | Participants anxious about how methadone can affect aging and overall health and chronic disease long-term. |
| Integrated services would be of significant benefit (45) | Participants expressed how integrated medical care with OUD treatment would be a benefit with aging and managing other chronic diseases. |
| Integrated services at OTP would be preferred (28) | Because of transportation issues and experienced stigma from outside healthcare providers, OTP would be an ideal place to receive all care. |
| Importance of coordinated care (12) | Healthcare providers are all at different places and do not communicate to help the patient. |
| Need for psychiatrist/psychologists at OTPs (10) | Regarding the need for better mental health providers at OTPs. |
| Importance of non-judgmental care (9) | Regarding not to feel judged or have to explain themselves. |
| OTP/methadone disrupts daily life or travel (15) | Regarding how the structure of the OTP prevents people from doing other daily activities and forces the day to center on getting to the OTP. |
| Benefits of methadone (12) | Regarding helping to function and be present for family. |
| Fear of withdrawal (7) | Regarding the reluctance to stop methadone due to symptoms of withdrawal. |
| Positive experience with PCP (19) | Some participants did have a good relationship with their PCP and relied on them for helping with chronic diseases. |
| Desire more health education with aging (7) | Regarding the need for more information about dealing with getting older. |
